# Personal relative deprivation associated with functional disorders via stress: An examination of fibromyalgia and gastrointestinal symptoms

**DOI:** 10.1371/journal.pone.0189666

**Published:** 2017-12-27

**Authors:** Shadi Beshai, Sanju Mishra, Sandeep Mishra, R. Nicholas Carleton

**Affiliations:** 1 Department of Psychology, University of Regina, Regina, SK, Canada; 2 Department of Medicine, Dalhousie University, Halifax, NS, Canada; 3 Department of Medicine, Division of Clinical Immunology & Allergy, Western University, London, ON, Canada; 4 Faculty of Business Administration, University of Regina, Regina, SK, Canada; University of Illinois at Urbana-Champaign, UNITED STATES

## Abstract

**Background:**

Personal relative deprivation is a negative social comparison process typified by self-comparison, negative appraisal, and resultant negative emotionality. Personal relative deprivation has been associated with poorer physical and mental health in several domains. It has been hypothesized that the deprivation-health link operates through a stress pathway. Stress has been specifically implicated in the onset and maintenance of functional disorders, including fibromyalgia and functional gastrointestinal disorders. Despite the theoretical links between personal deprivation, stress, and functional disorders, researchers have not assessed relationships between these variables.

**Methods:**

We recruited community participants (*n* = 517; 54.9% female) to examine whether personal relative deprivation can account for variance in fibromyalgia and functional gastrointestinal symptoms beyond known demographic correlates of physical health. We also examined whether the relationships between personal relative deprivation and functional disorder symptoms are mediated by stress.

**Results:**

Consistent with our hypotheses, personal relative deprivation accounted for symptom variance in fibromyalgia and functional gastrointestinal disorders beyond that accounted for by demographic variables alone. Further, self-reported stress was found to mediate relationships between personal relative deprivation and fibromyalgia and gastrointestinal symptoms.

**Conclusions:**

The current results support biopsychosocial models of physical health and suggest that, for patients presenting with functional disorders symptoms, a combination of biological and psychosocial interventions may be warranted.

## Introduction

Clinically significant mental health concerns contribute substantially to global disease burden [1, 2]. Mental health also has an enormous interactive influence on physical health [3]. Functional gastrointestinal disorders and fibromyalgia are chronic medical conditions that produce physiological symptoms in the absence of objective findings, and as such, involve both mental and physical components. The pathophysiology of such functional disorders remains unclear; nevertheless, mental health likely contributes to disease onset and chronicity of symptoms [4–6].

*Personal relative deprivation*—a negative social comparison process—has been recently evidenced as an important psychosocial influence for both physical and mental health [7–9]. In the current study, we examine whether the influence of personal relative deprivation extends to symptoms of functional disorders—namely, gastrointestinal distress and fibromyalgia—given that such disorders involve both mental and physiological components. We briefly review (1) the etiology of functional disorders, (2) evidence indicating that personal relative deprivation is linked with both physical and mental health, and (3) evidence that personal relative deprivation influences mental and physical health through a stress pathway. Finally, we present an empirical examination of the associations of personal relative deprivation, stress, and gastrointestinal and fibromyalgia symptoms.

### Functional disorders

Functional disorders are chronic medical conditions that produce physiological symptoms in the absence of identifiable organic pathology. The disorders produce substantial burden on healthcare resources, resulting in part from thorough clinical assessments necessary to rule out organic disease, and affected patients representing 30–50% of all outpatient visits [5, 10, 11]. Fibromyalgia and irritable bowel syndrome are the most commonly observed functional disorders in primary and subspecialty clinics [12, 13]. Fibromyalgia is a chronic condition associated with musculoskeletal pain, fatigue, and sleep difficulties [5]. Irritable bowel syndrome is a functional gastrointestinal disorder featuring chronic abdominal pain or discomfort in the setting of changed bowel habits [13]. Fibromyalgia and functional gastrointestinal disorder comorbidity is common, occurring in approximately 32.5% of patients [13].

Despite the comorbidity of fibromyalgia and functional gastrointestinal disorders, no unifying pathophysiology has been identified. Previous infection, altered pain response, stress, and mental health may all contribute to disease onset and symptom chronicity [14]. In particular, the role of mental health in physical disease has been extensively studied and is thought to play a prominent role in functional disorders. Psychiatric conditions are highly comorbid with functional conditions, but no causal relationship exists to date [14, 15]. The mechanism by which mental health contributes to functional disease is unclear, but chronic stress likely modulates this relationship [15].

### Relative deprivation, stress, and health

Previous theorizing has suggested that objective inequality motivates individual-level socioeconomic comparisons, which leads to stress, and eventually, poorer mental and physical health (reviewed in Wilkinson and Pickett [16]). Accordingly, stress is believed to be the driving proximate mechanism of the deleterious effects of personal relative deprivation. Stress has been implicated in the development and exacerbation of several physical and mental health conditions [17, 18]. For example, stress is an important predictor of depression [19] and anxiety [20].

The epidemiological literature has evidenced income inequality as associated with several mental health conditions (see [21] for review). Furthermore, an emergent body of literature has demonstrated that individual-level *subjective* perceptions of inequality, in addition to objective measures, predict poorer mental and physical health [7, 9, 22, 23]. The process of self-comparison, negative appraisal, and resultant negative emotionality is called *personal relative deprivation*. Accordingly, perceptions of personal status and social rank appear to be important predictors of mental and physical health outcomes.

Increasingly, epidemiological and empirical studies have demonstrated a pervasive link between personal relative deprivation, physical health, and mental health [7, 9, 24, 25]. However, no studies to-date have examined whether personal relative deprivation is associated with symptoms of functional disorders. Results of previous investigations (reviewed below) suggest that stress is a significant predictor of functional disorder symptoms. Given that the negative effects of personal relative deprivation are posited to operate through stress, it follows that deprivation may explain unique variance in functional disorders via a stress pathway.

Functional disorders have been linked to mental health generally. Aaron et al. [26] found that patients with fibromyalgia had a significantly greater number of lifetime psychiatric diagnoses than those without fibromyalgia. The overlap between mental health and functional disorders has also been investigated; for example, Kroenke et al. [27] found that the number of functional physical symptoms patients report in primary care is highly predictive of the presence of psychiatric disorders. In a review, Fadgyas-Standculete et al. [28] found that several psychiatric conditions, including depression, bipolar disorder, generalized anxiety, and schizophrenia, may exacerbate functional gastrointestinal disorders. Indeed, the link between mental health and functional disorders is so pervasive that Gatchel [29] suggested the adoption of biopsychosocial models and treatments in addressing these conditions (as opposed to a traditional purely physiological approach).

As mentioned, stress has been broadly linked to functional disorders [30, 31] and mental health [32] and represents one of the most robust negative predictors of mental health [19, 33]. Stress has also been implicated in the development and maintenance of fibromyalgia [34]; however, the evidence linking stress and functional gastrointestinal disorders is more robust (reviewed in [35–37]).

### The present study

Taken together, the evidence reviewed above suggests that (a) personal relative deprivation is an important predictor of psychological and physical distress; (b) personal relative deprivation may operate through increasing stress; and (c) stress is importantly implicated in several psychological and physical health conditions, particularly fibromyalgia and functional gastrointestinal disorders. In the present study, we examined whether personal relative deprivation operates through stress to influence symptoms of fibromyalgia and functional gastrointestinal disorder. Personal relative deprivation has been associated with both mental and physical disorders [7–9]. Given that functional disorders involve both mental and physical components, personal relative deprivation will likely explain unique variance in functional disorder symptomology. We predicted that (a) personal relative deprivation would be positively associated with self-reported symptoms of fibromyalgia and functional gastrointestinal disorders; (b) personal relative deprivation would account for symptom variance beyond known demographic correlates of mental and physical health (e.g., age, gender, education, marital status, and income); and (c) consistent with the relative deprivation hypothesis, stress would mediate the relationship between personal relative deprivation and functional disorder symptoms.

## Materials and methods

### Participants and procedure

Participants were recruited through the crowdsourcing website CrowdFlower. CrowdFlower and other crowdsourcing platforms have been used in several behavioral and clinical studies [38, 39]. CrowdFlower is an international alternative to the commonly used US-based crowdsourcing website Mechanical Turk (MTurk). MTurk has a proprietary participant pool, whereas CrowdFlower disseminates study tasks to various partner channels, each with their own workforce. There are over 5 million unique participants who use CrowdFlower [40]. Data collection occurred during March–April of 2016. A total of 517 participants (54.9% female) completed the study questionnaires. Sample demographic characteristics are summarized in Table 1. Participants were recruited from five English-speaking regions (Australia, Canada, New Zealand, United Kingdom, and United States of America). Study measures were presented in randomized order. All study participants were financially compensated for their participation. The research protocol for the present study was approved by the University of Regina’s Research Ethics Board (file #2015–199).

**Table 1:** Demographic characteristics.

	*n =* 517
Age: *M* (*SD*)	36.51 (11.86)
Sex: *n* (%)	
Female	284 (54.9)
Marital status	
Single	127 (24.6)
Dating	80 (15.5)
Married/Common-law	276 (53.4)
Separated/Divorced	15 (2.9)
Widowed Other	10 (1.9) 14 (2.7)
Education	
Secondary School or below	107 (20.7)
Some college/university	107 (20.7)
College/University	211 (40.8)
Post-Graduate/Professional School Other	62 (11.9) 32 (6.2)
Personal Annual Income Range	
Less than $20,000	127 (24.6)
$20,000 - $50,000	200 (38.7)
Over $50,000	192 (37.1)
PRDS-R: *M* (*SD*)*	18.85 (5.57)
DASS-Depression	13.74 (5.13)
DASS-Anxiety	12.50 (4.42)
DASS-Stress	14.21 (4.58)
FIQR	40.53 (19.97)
Gastro-Questionnaire	49.84 (14.26)

* PRDS—R = Personal Relative Deprivation Scale-Revised; DASS-Depression = Depression subscale of DASS-21; DASS-Anxiety = Anxiety subscale of DASS-21; DASS-Stress = Stress subscale of DASS-21; FIQR = Fibromyalgia Impact Questionnaire Revised; Gastro-Questionnaire = assessment of functional gastrointestinal disorder symptoms.

#### Measures

The *Personal Relative Deprivation Scale–Revised* (PRDS-R [22]) is a 5-item self-report measure designed to assess personal relative deprivation. The items are (1) “I feel deprived when I think about what I have compared to what other people like me have”; (2) “I feel privileged compared to other people like me”; (3) “I feel resentful when I see how prosperous other people like me seem to be”; (4) “When I compare what I have with what others like me have, I realize that I am quite well off”; (5) “I feel dissatisfied with what I have compared to what other people like me have”). Respondents were asked to rate such items on a 6-point scale ranging from 1 (*strongly disagree*) to 6 (*strongly agree*). The scale appears reliable and internally consistent [22]. The PRDS-R has been associated with diverse outcomes including depression symptoms, anxiety symptoms, physical health problems, gambling urges, problem gambling, delayed discounting, antisocial behaviors, criminal conduct, and traits associated with risk-taking [7–9, 22, 23]. In the current study, the PRDS-R items produced an internal consistency of *α* = .81.

*Depression Anxiety Stress Scale* (DASS-21; [41]). The DASS-21 is a 21-item self-report measure that consists of three subscales: Depression, Anxiety, and Stress, each scored on 7 items. Each of the three subscales assessed the severity of depression (e.g., “I couldn’t seem to experience any positive feeling at all”), anxiety (e.g., “I felt that I was close to panic”), and stress (e.g., “I found it hard to wind down") symptoms on a four-point scale from 0 (*did not apply to me at all*) to 3 (*applied to me very much*, *or most of the time*) over the past week. Accordingly, scores on each of the three subscales can range from 0–21, with a range for total scores of 0–63, with higher scores being indicative of greater distress. A cut-off of 10 or greater on either the depression or anxiety subscales of the DASS-21 appears to have adequate sensitivity and specificity in diagnosing depression or anxiety [42]. The DASS-21 has demonstrably excellent psychometric properties among clinical and community samples [43], and has been validated among community and clinical samples [44, 45]. In the current study, the depression, anxiety, and stress subscale items of the DASS-21 produced internal consistencies of *α* = .92, .87, and .89, respectively.

The Revised Fibromyalgia Impact Questionnaire (FIQR; [46]) is a commonly used 21-item self-report instrument that assesses symptoms of fibromyalgia (e.g., pain, fatigue, stiffness, poor sleep, depression) and their impact on daily living over the past week. Symptom severity items (10 in total) are rated on an 11-point Likert scale, from 0 (*no symptoms*) to 10 (*extreme/severe symptoms*), and higher scores on such items are indicative of greater fibromyalgia severity (range of 0 to 100). The FIQR has been extensively validated in clinical trials (e.g., [47]). In the current study, the FIQR items produced an internal consistency of *α* = .89.

The Gastro-Questionnaire [48] is a 27-item instrument designed to assess frequency and severity of functional gastrointestinal symptoms over the last 12 months. One of the items (item 4, Nausea) was erroneously omitted from the electronic survey; therefore, the current study used 26 of the original 27 items. Each of the items was scored on a 4-point scale, from 1 (*not at all*) to 4 (ne*arly always*), with higher scores indicative of higher severity. The scale has been validated [48], and has been used among clinical and general population samples [49]. In the current study, items of the Gastro-Questionnaire produced an internal reliability of *α* = .94.

### Data analysis overview

All analyses were performed on SPSS v. 23.0 (IBM, Chicago, USA). Missing data were minimal (< 5%); accordingly, no imputation methods were used and cases with missing values were not included in the analyses. An alpha level of .05 was used to determine significance for all analyses. Pearson correlations were calculated to examine the relationship between scores on the PRDS-R, Depression, Anxiety, and Stress Subscales of the DASS-21, FIQR, and Gastro-Questionnaire. We performed two hierarchical regression analyses to examine whether personal relative deprivation (measured by PRDS-R) significantly predicted functional gastrointestinal disorder (Gastro-Questionnaire) and fibromyalgia (FIQR) symptoms, beyond what is accounted for by demographic variables. Specifically, the first of two planned hierarchical regression analyses examined whether PRDS-R scores significantly predicted scores on the FIQR beyond age, gender, marital status, education, and income, all known demographic correlates of health. Demographic variables were entered in the first block and PRDS-R scores were entered in the second block. Scores on the FIQR were used as the criterion (dependent) variable in each of the three analyses. The second hierarchical regression analysis examined whether PRDS-R scores significantly predicted Gastro-Questionnaire scores beyond age, gender, marital status, education, and income. Demographic variables were entered in the first block and PRDS-R scores were entered in the second block. Gastro-Questionnaire scores were used as the criterion (dependent) variable.

We conducted two mediational analyses using Preacher and Hayes [50, 51] bootstrapping methodology (10,000 resamples) to examine whether stress (DASS-Stress Subscale) mediated the relationships of personal relative deprivation (PRDS-R) and each of the functional disorders symptom scales (Gastro-Questionnaire and FIQR). Finally, we conducted several exploratory, ancillary t-test analyses to examine whether individuals who self-reported diagnoses of functional disorders differed on key measures of interest.

## Results and discussion

### Personal relative deprivation and fibromyalgia symptoms

Total scores on the PRDS-R were significantly and positively associated with scores on the FIQR, *r* = .30, *p* < .001. The full correlation matrix is presented in Table 2. Further, results from the first hierarchical regression analysis indicated PRDS-R scores were predictive of scores on the FIQR beyond what can be explained by demographics. The regression analyses results are summarized in Table 3.

**Table 2:** Correlation matrix for study measures.

Measure	1	2	3	4	5
1. PRDS-R^a^	-				
2. DASS-Depression	.49**	-			
3. DASS-Anxiety	.34**	.71**	-		
4. DASS-Stress	.37**	.78**	.76**	-	
5. FIQR	.30**	.56**	.60**	.58**	-
6. Gastro-Questionnaire	.19**	.45**	.54**	.49**	.61**

*a–*PRDS—R = Personal Relative Deprivation Scale-Revised; DASS-Depression = Depression subscale of DASS-21; DASS-Anxiety = Anxiety subscale of DASS-21; DASS-Stress = Stress subscale of DASS-21; FIQR = Fibromyalgia Impact Questionnaire Revised; Gastro-Questionnaire = assessment of functional gastrointestinal disorder symptoms.

** = *p* < .001

**Table 3:** Results of four regression analyses of PRDS-R scores as predicting scores on outcome measures beyond variance contributed by key demographic variables.

Outcome: FIQR^a^	*B*	*SE*	*β*	*t*
Step 1: *R* = .20, *R*^*2*^ = .04				
Age	-.06	.08	-.03	0.47
Gender	6.96	1.8	.18	3.84**
Marital Status	-.01	.50	-.00	1.00
Education	-.88	.62	-.07	-1.43
Personal Income	-.18	.36	-.24	-0.51
Step 2: *R* = .37, *R*^*2*^ = .14, ***Δ****R2* = .10**				
Age	-.02	.07	-.01	-0.23
Gender	7.16	1.72	.18	4.16**
Marital Status	.37	.47	.04	0.77
Education	-.87	.58	-.07	-1.48
Personal Income	.29	.35	.04	0.83
PRDS-R	1.15	.15	.33	7.49**
**Outcome: Gastro-Questionnaire**				
Step 1: *R* = .06, *R*^*2*^ = .003				
Age	-.06	.06	-.05	-1.05
Gender	.88	1.29	.03	0.68
Marital Status	-.12	.35	-.02	-0.34
Education	.03	.44	.00	0.07
Personal Income	.12	.26	.02	0.46
Step 2: *R* = .22, *R*^*2*^ = .05, ***Δ****R*^*2*^ = .04**				
Age	-.04	.05	-.03	-0.70
Gender	.99	1.26	.04	0.78
Marital Status	.04	.35	.005	0.11
Education	.04	.43	.005	0.10
Personal Income	.33	.25	.06	1.31
PRDS-R	.55	.11	.22	4.85**

*a–*PRDS—R = Personal Relative Deprivation Scale-Revised; DASS-Depression = Depression subscale of DASS-21; DASS-Anxiety = Anxiety subscale of DASS-21; DASS-Stress = Stress subscale of DASS-21; FIQR = Fibromyalgia Impact Questionnaire Revised; Gastro-Questionnaire = assessment of functional gastrointestinal disorder symptoms.

** = *p* < .001

We conducted a mediational analysis using bootstrapping methodology [50, 51] to examine whether stress (DASS-Stress) significantly mediated the relationships of personal relative deprivation (PRDS-R) and fibromyalgia symptoms (i.e., FIQR scores). Summary of this analysis is provided in Table 4. The results indicated that stress partially mediated the relationship of personal relative deprivation and fibromyalgia symptoms as assessed by the FIQR (see Table 4).

**Table 4:** Two meditation analyses examining whether stress (DASS-Stress scores) mediates relationships of personal relative deprivation (PRDS-R scores) and scores on outcome measures.

Measure	Path A *b* (SE)	Path B *b* (SE)	Path C *b* (SE)	Path C’ *b* (SE)	Indirect effect *b* (95% bias-corrected CI)
FIQR	.31** (.03)	2.36** (.17)	1.10** (.15)	.37* (.14)	.73 (.56, .92)
Gastro-Questionnaire	.31**(.03)	1.48** (.13)	.50** (.11)	.05 (.11)	.46 (34, .60)

*Note*. FIQR = Fibromyalgia Impact Questionnaire Revised; Gastro-Questionnaire = assessment of functional gastrointestinal disorder symptoms.

* = *p* < .01

** = *p* < .001

#### Ancillary analyses

A total of 179 participants reported being diagnosed with fibromyalgia. These participants scored significantly higher (*M* = 48.67, *SD* = 18.78) on the FIQR compared to those who did not report a diagnosis (*M* = 36.05, *SD* = 19.19), *t*(516) = 7.11, *p* < .001, *d* = 0.66. Additionally, participants who reported a diagnosis of fibromyalgia scored significantly higher (*M* = 19.72, *SD* = 4.88) than those who did not report such diagnosis (*M* = 18.37, *SD* = 5.86) on the PRDS-R, *t*(516) = 9.56, *p* < .001, *d* = 0.25.

### Personal relative deprivation and functional gastrointestinal symptoms

PRDS-R total scores were significantly and positively associated with scores on the Gastro-Questionnaire. Correlation coefficients for study measures are summarized in Table 2. Results of the planned hierarchical regression revealed that personal relative deprivation (PRDS-R Scores) were significantly predictive of functional gastrointestinal disorder symptoms (Gastro-Questionnaire) beyond what is predicted by demographics alone. The regression results are summarized in Table 3.

**Fig 1:**
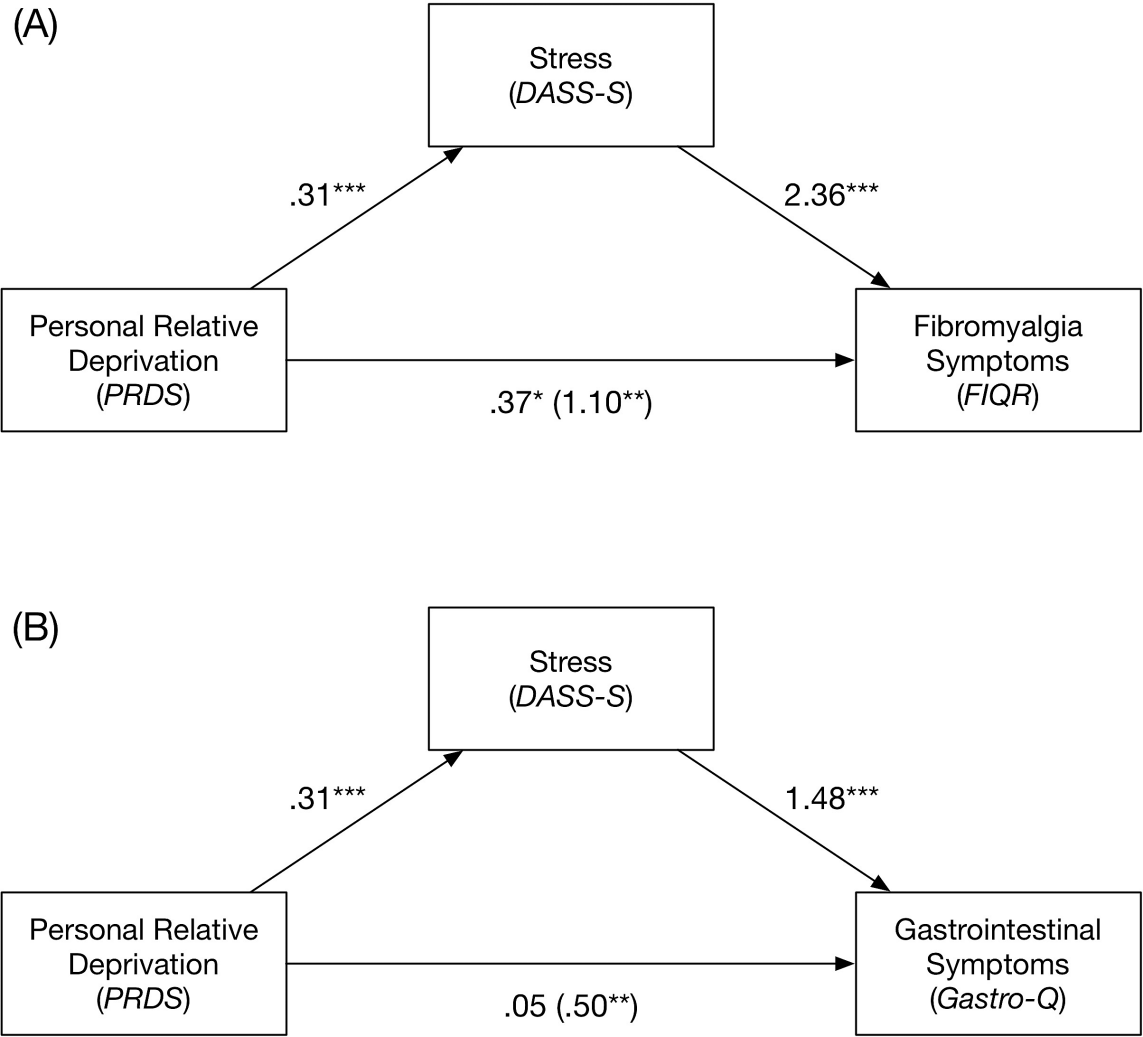
Bootstrapped mediation models of the mediating effect of stress in the relationships of personal relative deprivation and fibromyalgia symptoms (A), and personal relative deprivation and gastrointestinal symptoms (B). Stress partially mediated the relationship of personal relative deprivation and fibromyalgia symptoms, and fully mediated the relationship of personal relative deprivation and gastrointestinal symptoms.

We also conducted a planned mediation analysis using [51] PROCESS plugin for SPSS. PROCESS uses a bootstrapping (10,000 resamples) technique to examine mediation models [50]. The mediation analysis was used to examine whether stress (DASS Stress Subscale) mediated the relationship between personal relative deprivation (PRDS-R) and gastrointestinal symptoms (Gastro-Questionnaire). Results of this analysis are summarized in Table 4. Stress (DASS Stress Subscale) fully mediated the relationship between personal relative deprivation and gastrointestinal symptoms (see Fig 1).

#### Ancillary analyses

A total of 274 participants reported being diagnosed with the functional gastrointestinal disorder irritable bowel syndrome. Participants who reported a diagnosis (*M* = 53.46; *SD* = 13.24) scored significantly higher on the Gastro-Questionnaire than participants who did not (*M* = 45.77; *SD* = 14.28), *t*(516) = 6.36, *p* <. 001, *d* = 0.56. There were no significant difference between those reporting a diagnosis of irritable bowel syndrome (*M* = 18.67; *SD* = 5.62) and those who did not (*M* = 19.05; *SD* = 5.51) on the PRDS-R, *t*(516) = -0.77, *p* = .44, *d* = .07. Further, participants reporting a diagnosis of gluten sensitivity (*n* = 65) also scored significantly higher on the Gastro-Questionnaire (*M* = 57.21; *SD* = 14.66) than those not reporting this diagnosis (*M* = 48.78; *SD* = 13.90), *t*(516) = 4.55, *p* < .001, *d* = 0.40. There were no significant differences between participants reporting a diagnosis of gluten sensitivity (*M* = 19.42; *SD* = 4.97) and those who did not report this diagnosis (*M* = 18.76; *SD* = 5.64) on PRDS-R scores, *t*(516) = 0.38, *p* = .38, *d* = .03.

Consistent with our hypotheses, results indicated that personal relative deprivation is associated with self-reported symptoms of both physical conditions, and that relative deprivation can explain variance in such symptoms beyond what can be explained by demographic variables alone. We also demonstrated that personal perceptions of deprivation may contribute to symptoms of functional disorders through increased stress. Stress partially mediated the association of relative deprivation and fibromyalgia symptoms, and fully mediated the relationship of personal relative deprivation and functional gastrointestinal symptoms.

Recently, researchers have shown that personal relative deprivation is linked to various indices of mental health [7–9]. For example, Beshai et al. [7] demonstrated that scores on a personal relative deprivation measure were positively and consistently correlated with depression symptoms, as measured by several validated measures of the condition. Beshai and colleagues also found that negative self-referent thoughts (e.g., “I am no good”) fully mediated the relationship between personal relative deprivation and depression symptoms, suggesting that negative self-referent thoughts may be the proximate mechanism by which personal relative deprivation may increase depression. Further, personal relative deprivation has been associated with externalizing and problem social behaviours, such as gambling and risk-taking more generally [23, 52].

Our results provide further evidence that psychosocial inputs have important influence on functional disorders. The current results are consistent with some recent studies indicating that psychological interventions have some success in alleviating functional disorder symptoms. Kaptchuk et al. [53] found that a group of patients receiving placebo pills without deception evidenced significantly improved irritable bowel syndrome symptoms when compared to a group that received no treatment. Similarly, a recent study of functional dyspepsia patients (i.e., those with chronic indigestion) showed that mirtazapine, an antidepressant, significantly improved quality of life [54]. Further, and in support of the psychosocial hypothesis of functional disorders, several studies have demonstrated that evidence-based psychological interventions such as cognitive therapy are successful in reducing irritable bowel and fibromyalgia symptoms [55–57]. Larger clinical studies are necessary to determine whether certain patients (e.g., those with heightened personal relative deprivation) may benefit from targeted psychosocial interventions to optimize their functional symptoms.

Psychosocial factors appear to have a clear and important influence on functional disorders [58, 59]; nevertheless, some recent studies suggest that some subtypes of functional disorders have a stronger biological basis than previously identified [14, 60, 61]. For example, in a recent study, Pridgen et al. [62] found that individuals with fibromyalgia who were randomized to receive famciclovir and celecoxib (i.e., a treatment used to remediate viral herpes) experienced significantly improved symptoms when compared against a control group not receiving the treatment. The results may indicate a subtype of fibromyalgia that is exacerbated by persistent viral infections. Similarly, increasing evidence suggests irritable bowel syndrome has differing pathophysiology based on different symptom profiles [63]. Further research is necessary to better understand the interaction of physiological and psychosocial inputs in the etiology of functional disorders, especially considering recently discovered subtypes.

The current study is the first to examine the degree and mechanism through which personal relative deprivation is associated with symptoms of functional disorders, such as fibromyalgia and functional gastrointestinal disorder. The results extend current knowledge in several important ways. First, although clinical diagnoses of the targeted functional disorders could not be ascertained, we used common and validated self-report measures of symptom severity. Second, our sample was large and diverse, suggesting the study was sufficiently powered to test our hypotheses. Third, we did not test our predictions among an analogue convenience (student) sample, which would have limited generalizability of our results.

The present study also has several limitations that provide directions for future research. First, participants were recruited using an online crowdsourcing platform and never assessed clinically; accordingly, the contribution of organic disease could not be assessed. Second, crowdsourced samples are not entirely representative of the general population, as they tend to be younger, more educated, and present with a particular clinical profile [39]. Third, we used a cross-sectional study design making causal inferences indefensible. We cannot know whether relative deprivation is cause or consequence of functional disorder symptoms, or whether all are influenced by a third confounding factor. Additionally, the functional disorder self-report measures we used are robust and clinically validated measures; however, the surveys were validated with now outdated diagnostic criteria [64, 65]. In future studies, researchers should use the American College of Rheumatology 2010 criteria for diagnosis of fibromyalgia and Rome IV criteria for diagnosis of functional gastrointestinal disorders [61, 66]. Finally, and although we demonstrated that increased stress is a potential mediator in the relationship of personal relative deprivation and functional disorder symptoms, we did not examine factors that fuel this stress specifically, such as work or relationship strain, financial difficulties, and others.

Future studies should examine whether personal relative deprivation can prospectively predict symptoms of functional disorders. There is a large degree of multicollinearity between mental health (e.g., depression and anxiety symptoms), functional symptoms, and relative deprivation; as such, future studies should elucidate the unique contribution of personal relative deprivation on physical health. Finally, despite the proliferation of evidence-based psychosocial interventions to reduce stress (e.g., Mindfulness-Based Stress Reduction; [67]), such interventions have not targeted functional disorder symptoms and may be promising solutions.

## Conclusions

The current results further support biopsychosocial models of physical health symptoms. Personal relative deprivation significantly predicted functional disorder symptoms. The relationship appears maintained through the hypothesized mechanism of increased stress. The results further highlight the need to address functional disorders using a biopsychosocial approach, rather than purely biological approaches. For patients, supplementing biological treatments with evidence-based psychotherapy (e.g., low intensity and self-administered cognitive therapy; [68]) may be particularly promising.

## Supporting information

S1 Data Data from Beshai et al. Relative Deprivation and Functional Disorders.
(SAV)
